# Silencing PCBP2 normalizes desmoplastic stroma and improves the antitumor activity of chemotherapy in pancreatic cancer

**DOI:** 10.7150/thno.53102

**Published:** 2021-01-01

**Authors:** Yuanke Li, Zhen Zhao, Chien-Yu Lin, Yanli Liu, Kevin F. Staveley-OCarroll, Guangfu Li, Kun Cheng

**Affiliations:** 1 Division of Pharmacology and Pharmaceutical Sciences, School of Pharmacy, University of Missouri-Kansas City, 2464 Charlotte Street, Kansas City, MO 64108, USA; 2 Department of Surgery, School of Medicine, University of Missouri, One Hospital Drive, Columbia, MO 65212

**Keywords:** PCBP2, siRNA, pancreatic cancer, tumor stroma, nanocomplex

## Abstract

**Rationale:** Dense desmoplastic stroma is a fundamental characteristic of pancreatic ductal adenocarcinoma (PDAC) and comprises up to 80% of the tumor mass. Type I collagen is the major component of the extracellular matrix (ECM), which acts as a barrier to impede the delivery of drugs into the tumor microenvironment. While the strategy to deplete PDAC stroma has failed in clinical trials, normalization of the stroma to allow chemotherapy to kill the tumor cells in the “nest” could be a promising strategy for PDAC therapy. We hypothesize that silencing the poly(rC)-binding protein 2 (αCP2, encoded by the PCBP2 gene) leads to the destabilization and normalization of type I collagen in the PDAC stroma.

**Methods:** We develop a micro-flow mixing method to fabricate a peptide-based core-stabilized PCBP2 siRNA nanocomplex to reverse the accumulation of type I collagen in PDAC tumor stroma. Various *in vitro* studies were performed to evaluate the silencing activity, cellular uptake, serum stability, and tumor penetration of the PCBP2 siRNA nanocomplex. We also investigated the penetration of small molecules in stroma-rich pancreatic cancer spheroids after the treatment with the PCBP2 siRNA nanocomplex. The anti-tumor activity of the PCBP2 siRNA nanocomplex and its combination with gemcitabine was evaluated in an orthotopic stroma-rich pancreatic cancer mouse model.

**Results:** Silencing the PCBP2 gene using siRNA reverses the accumulation of type I collagen in human pancreatic stellate cells (PSCs) and mouse NIH 3T3 fibroblast cells. The siRNA nanocomplex significantly reduces ECM production and enhances drug penetration through desmoplastic tumor stroma. The combination of gemcitabine with the PCBP2 siRNA nanocomplex markedly suppresses the tumor progression in a desmoplastic PDAC orthotopic mouse model.

**Conclusion:** This approach provides a new therapeutic avenue to improve the antitumor efficacy of PDAC therapies by normalizing tumor stroma using the PCBP2 siRNA nanocomplex.

## Introduction

Pancreatic ductal adenocarcinoma (PDAC) is one of the leading causes of cancer-related mortality in the world 1. The 5-year relative survival rate is very low, approximately 9% for all stages combined 2. The incidence of PDAC is increasing, and it is projected to become the second most common cause of cancer death in the United States by 2030, overtaking colorectal cancer 3. In contrast to the sustained and steady increase in survival for patients with most cancers, the survival of patients with PDAC has not improved substantially over the past 40 years 4, 5. Therefore, the development of a novel and effective treatment for PDAC is urgently needed.

Desmoplasia, the abundant fibrotic stroma, is one of the most prominent characteristics of PDAC and comprises up to 80% of the tumor mass 4, 6. In the stromal microenvironment, activated pancreatic stellate cells (PSCs) transform from a quiescent state into a myofibroblast-like phenotype and express a large amount of extracellular matrix (ECM) proteins, including collagens, fibronectins and laminins 7, 8. Type I collagen proteins are the most abundant and main component of the ECM in PDAC and are responsible for the major desmoplastic reaction 9, 10. High levels of type I collagen are associated with a low survival rate for patients with PDAC 8, 9. Type I collagen promotes the proliferation and migration of PDAC cells and inhibits apoptotic cells by binding to integrin 9, 11. In addition, activated PSCs and the deposition of ECM generate a high level of solid stress and tissue interstitial fluid pressure, which both cause compression and dysfunction of the vasculature 7, 12, 13. This expanded fibrotic stroma and dysfunctional vasculature around PDAC cells impede drug penetration and reduce the therapeutic efficacy 7, 8, 13. In theory, stromal depletion therapies may improve the survival, but clinical trials employing strategies designed to deplete the stroma have failed 14, 15. The stroma acts not only as a barrier for drug delivery but also a protective defense mechanism to restrain the growth of PDAC tumors 4, 15. Consequently, the complete depletion of the stroma leads to more aggressive tumor cells and a poor survival rate 7, 14. Therefore, the development of approaches that normalize the tumor stroma combined with antitumor drugs to kill the tumor cells in the “nest” is a promising strategy for PDAC therapy.

Type I collagen, a heterotrimer, consists of two α1(I) and one α2(I) polypeptide chains 16. The accumulation of type I collagen proteins in the liver was attributed to an increase in the mRNA level mediated by increased mRNA stability. The stabilization of type I collagen α1(I) is mediated by the binding of the poly(rC)-binding protein 2 (αCP2, encoded by the PCBP2 gene) to the 3'-UTR regions of the type I collagen α1(I) mRNA 17, 18. This post-transcriptional mechanism was identified in NIH/3T3 fibroblast cells and activated hepatic stellate cells (HSCs), but not quiescent cells 19-21. The PSCs and HSCs display a great degree of similarity in morphological and functional characteristics and both are derived from a common fibroblast lineage 22. In a study, 23,000-feature whole genome oligonucleotide micro-arrays were used to compare the expression profiling of PSCs and primary human HSCs. The results demonstrated only 29 genes were differentially expressed between PSCs and HSCs 23. We, therefore, postulated that the αCP2 protein also increases the stability of the type I collagen α1(I) mRNA in activated PSCs. In this study, we aim to normalize the accumulation of type I collagen in the PDAC stroma by silencing the expression of the PCBP2 gene with siRNAs.

Late in the last century, RNA interference (RNAi) became a promising biological tool for treating various diseases, including genetic disorders, viral infection, and cancers 24-26. Due to the fast degradation and clearance in the body after injection and poor cellular uptake and lysosomal escape, an efficient system designed to deliver the siRNA to the desired site remains a major challenge for siRNA applications in the clinic 27, 28. Various siRNA delivery systems, including non-viral and viral vectors, have been explored to overcome these barriers 29-31. Although viral vectors function as siRNA carriers with high transfection and stable gene silencing efficiencies, their application poses problems due to concerns regarding toxicity, such as immunogenicity and inflammation 32. Despite the recent success of two siRNA drugs approved by the Food and Drug Administration (FDA), both of them deliver the siRNA to the liver. Delivery of siRNAs to non-hepatic tissues remains a challenge. We previously developed a series of siRNA delivery systems by conjugating cholesterol to the N-terminus of short peptides containing natural amino acids HHHKKHHHKK, which is fully biocompatible and can be easily synthesized. The cholesterol-peptide spontaneously forms a micelle-like structure and condenses siRNAs into nanocomplexes in aqueous solutions 32. Here, we include a cathepsin B-labile dipeptide (Val-Cit) between the cholesterol and hydrophilic peptides to improve the escape of the siRNA after endocytosis. Although the micelles possess a low critical micellar concentration (CMC), high stability is difficult to maintain through self-assembly alone when they are injected into the blood 33, 34. One of the most desirable approaches to improve the structural stability of micelles is cross-linking of the backbones to maintain the self-assembled nanostructure. We, therefore, incorporate a cysteine into the micelles to increase their stability by the formation of disulfide bonds, which will be cleaved by glutathione inside tumor cells to release the siRNA 35.

As illustrated in **Scheme I**, we design three cholesterol modified peptides (Figure S1), cholesterol-peptide (CP), cholesterol-peptide-cysteine (CPC), and cholesterol-cysteine-peptide (CCP) to condense the PCBP2 siRNA to form nanocomplexes. We investigate the effect of the PCBP2 siRNA on reversing the accumulation of type I collagen using the siRNA nanocomplexes (**Scheme I**). Normalization of the ECM dramatically increased the drug penetration into the stroma-rich three-dimensional (3D) tumor spheroids. Combination of gemcitabine with the siRNA nanocomplex markedly suppresses the tumor progression in a desmoplastic PDAC orthotopic mouse model.

## Material and Methods

### Materials

Lipofectamine® RNAiMAX and mouse PCBP2 siRNA (sense strand sequence: 5'-GUCAGUGUGGCUCUCUUAUtt-3') was ordered from Invitrogen (Carlsbad, CA). Human PCBP2 siRNA (sense strand sequence: 5'-GUCAGUGUGGCUCUCUCAUtt-3') was ordered from Dharmacon Inc. (Lafayette, CO). Mouse serum was purchased from BD Biosciences (San Jose, CA). Cholesterol-Val-Cit-CHHHKKHHHKK and Cholesterol-Val-Cit-HHHKKHHHKKC were ordered from United BioSystems Inc. (Herndon, VA).

### Preparation of the siRNA nanocomplexes

The cholesteryl peptide/siRNA nanocomplexes were prepared using a micro-flow mixing device as depicted in **Figure 2B**. siRNA was diluted to 2 μM in diethylpyrocarbonate (DEPC)-treated water. Similarly, cholesteryl peptides were diluted with DEPC-treated water to the final concentrations corresponding to various N/P ratios. Equal volumes of cholesteryl peptides and siRNA solutions were loaded into the two syringes (Hamilton, Reno, NV), which are mounted on a syringe pump (Chemyx, Inc., Stafford, TX). The two solutions were pumped at various flow rates (0.05, 0.1, 0.5, and 1.0 mL/min) and mixed in a Micro Static Mixing Tee (IDEX Health & Science, Oak Harbor, WA) to form the nanocomplexes. The internal geometry of the tee allows efficient mixing of two fluid streams. The final nanocomplexes were stabilized at room temperature for 15 mins before being analyzed in subsequent experiments. For “bulk mixing”, the solutions were mixed with a pipette. The particle size and zeta potential of each nanocomplex were evaluated using dynamic light scattering (DLS). The morphology of the nanocomplexes was examined using transmission electron microscopy (TEM). The cholesteryl peptide/siRNA nanocomplexes were incubated with 50% mouse serum to study the serum stability for different time intervals at 37 ºC. Heparin (40 μM) was incubated with siRNA nanocomplexes for 10 min to dissociate the siRNA from the nanocomplex. siRNA nanocomplexes mixed with serum was analyzed by electrophoresis in a 1% agarose gel, and siRNA was visualized by staining with GelRed.

The release study of siRNA from the nanocomplex was performed as previously reported with modification 36, 37. Cathepsin B enzyme extracted from human liver (Sigma-Aldrich, MO) was first activated at room temperature for 15 min 36. The CCP/siRNA nanocomplex (50 uM) was then treated with Cathepsin B (12.5 μM) and/or glutathione (10 mM) at 37 ºC for 6 h. Release of the siRNA was determined using a gel retardation assay on a 1% agarose gel.

### Cell culture

PANC-1 cells and NIH 3T3 cells were purchased from the American Type Culture Collection (Manassas, VA) and cultured in Dulbecco's Modified Eagle Medium (DMEM). Primary human pancreatic stellate cells (PSCs) were purchased from iXCells Biotechnologies and cultured in the stellate cell growth medium (San Diego, CA). Luciferase-pcDNA3 plasmid (Addgene, Watertown, MA) was transfected to PANC-1 cells to generate the stable cell line PANC-1/Luc.

### *In vitro* silencing activity

Cells were transfected using the method described in previous studies 21, 32. Briefly, human PSCs (8×10^4^ cells per well) or NIH 3T3 cells (1×10^5^ cells per well) were cultured in a 6-well plate for overnight before transfection. The PCBP2 siRNA was complexed either with Lipofectamine® RNAiMAX according to the manufacturer's protocol or with cholesteryl peptides as described above in Opti-MEM. The cells were washed with fresh DPBS, followed by addition of the transfection mixture at a final siRNA concentration of 100 nM. A scrambled siRNA was used as negative control. After transfection for 6 or 24 h, total RNA was isolated with a GenElute^TM^ Mammalian Total RNA Miniprep Kit (Sigma-Aldrich, St. Louis, MO) and then analyzed with a real-time PCR detection system (Bio-Rad, Hercules, CA) using the iTaq™ Universal SYBR^®^ Green One-Step Kit. The relative mRNA levels in treated samples were quantified using the comparative threshold (Ct) method with 18s as the reference.

For western blotting, human PSCs (1×10^5^ cells per well) or NIH 3T3 cells (2×10^5^ cells per well) were cultured in a 6-well plate for 12 h and then incubated with the PCBP2 siRNA or scrambled siRNA complexed with Lipofectamine® RNAiMAX for 24 h. The cells were collected and then re-cultured in a 6-well plate as mentioned above, followed by the second transfection. After three transfections, total protein were isolated for blotting with an anti-type I collagen antibody (Novus Biologicals LLC, Littleton, CO) and anti-PCBP2 antibody (GenWay Biotech, San Diego, CA) as previously reported. β-actin was used as an internal control 30. Three independent experiments were performed for each western blot.

For immunofluorescence staining, NIH 3T3 cells (2×10^5^ cells per well in 6-well plates) were transfected with siRNA nanocomplexes for 24 h. The cells that were transfected with the scrambled siRNA mixed with Lipofectamine® RNAiMAX served as a control. After three transfections, the cells were collected and seeded in 96-well clear bottom black plates at a density of 5000 cells/well. After incubation at 37ºC for 12 h, the cells were fixed with 4% paraformaldehyde for 20 min, blocked with 3% bovine serum albumin (BSA) for 2 h, incubated with type I collagen or ⍺CP2 antibody, and stained with Alexa Fluor 488 conjugated secondary antibody. The expression of type I collagen and ⍺CP2 was evaluated under a fluorescence microscope (Leica DMI3000B, Germany).

### Analysis of cellular uptake using flow cytometry and confocal microscopy

For the flow cytometry analysis, NIH 3T3 cells (1×10^5^ cells per well) were cultured into 6-well plates for 12 h. The medium was then replaced with Opti-MEM containing free Cy5-siRNA or Cy5-siRNA nanocomplexes (CP/Cy5-siRNA, CPC/Cy5-siRNA or CCP/Cy5-siRNA) at an N/P ratio of 5/1. After incubation for 2 h or 4 h, the cells were trypsinized and re-suspended in DPBS for flowcytometry analysis.

For confocal microscopy, NIH 3T3 cells (6 ×10^5^ cells) were seeded into a 4-well Nunc™ Lab-Tek™ Chambered Coverglass and cultured for 12 h, followed by incubation with free Cy5-siRNA or Cy5-siRNA nanocomplexes (CP/Cy5-siRNA, CPC/ Cy5-siRNA or CCP/ Cy5-siRNA) at an N/P ratio of 5/1. After a 4 h incubation, the cells were washed with DPBS and incubated with LysoTracker Red DND-99 (Invitrogen, Carlsbad, CA), followed by fixation with 4% paraformaldehyde and treatment with DAPI for nuclear staining. The cells were then imaged with a confocal microscope (Leica TCS SP5, Wetzlar, Germany).

### *In vitro* cytotoxicity study

PANC-1 cells or NIH 3T3 cells were seeded in black 96-well plates at a density of 5000 cells/well. After 12 h, the medium was replaced with Opti-MEM containing CP, CPC or CCP/PCBP2 siRNA nanocomplexes (N/P ratio of 5/1) at an siRNA concentration of 100 nM. After a 24 h incubation, cell viability was detected with CellTiter-Glo Kit (Promega, Madison, WI). Untreated cells served as the negative control, and the cells treated with Triton X-100 served as the positive control.

### Penetration of the siRNA nanocomplexes in stroma-rich three-dimensional (3D) tumor spheroids

The stroma-rich 3D tumor spheroids were prepared as previously described 38. Briefly, a mixture of PANC-1 tumor cells and NIH 3T3 fibroblast cells at a ratio of 2:1 was suspended in Spheroid Formation ECM (Amsbio, Cambridge, MA) according to the manufacturer's instructions and then added to the Corning 96-well ultralow attachment microplates (Pittsburgh, PA). The cells were incubated at 37ºC to form stroma-rich tumor spheroids. After 7 days of incubation, the stroma-rich tumor spheroids were incubated with Cy5-siRNA nanocomplexes at 37ºC for 2 h, followed by washes with DPBS and fixation with 4% paraformaldehyde. The tumor spheroids were then visualized using a confocal microscope to evaluate the penetration of the siRNA nanocomplexes.

### Penetration of small molecules into stroma-rich 3D tumor spheroids after treatment with the PCBP2 siRNA

The stroma-rich 3D tumor spheroids were prepared as described above on Day 1, and medium was replaced with Opti-MEM containing CCP/PCBP2 siRNA or CCP/scrambled siRNA nanocomplexes (N/P ratio of 5/1) at an siRNA concentration of 100 nM on Day 4. After an incubation for 24 h, the Opti-MEM was replaced with complete DMEM on Day 5. Similarly, stroma-rich tumor spheroids were transfected with 100 nM CCP/PCBP2 siRNA or CCP/scrambled siRNA two additional times on Day 6 and Day 8. After treatment with the CCP/siRNA nanocomplexes for a total of three times, the stroma-rich tumor spheroids were incubated with 10 μg/mL Hoechst 33258 at 37 ºC for 2 h or 4 h, followed by visualization using a confocal microscope to evaluate the penetration of Hoechst 33258 in stroma-rich tumor spheroids. Hoechst 33258 is a fluorescence probe that has been widely used in penetration studies to mimic small molecule drugs. The penetration depth of Hoechst 33258 in tumor spheroids was determined. The percentage of the penetration depth of Hoechst 33258 in tumor spheroids was calculated by dividing the penetration depth by the radius of the spheroids.

### Antitumor activity of the siRNA nanocomplexes in an orthotopic model of desmoplastic pancreatic tumor

The animal protocol was approved by the Institutional Animal Care and Use Committee (IACUC) of the University of Missouri-Kansas City. A mixture of 8 × 10^5^ PANC-1/Luc tumor cells and 4 × 10^5^ NIH 3T3 fibroblast cells was orthotopically injected into the tail of the pancreas in nude mice to establish the orthotopic desmoplastic tumor model, and the mice were randomly divided into four groups (n = 8) on Day 0. Group one: intravenous injection of saline on Days 10, 12, 14, 17, 19, 21 and 28. Group two: intravenous injection of saline on Days 10, 12, 17, and 19 plus an intravenous injection of gemcitabine (10 mg/kg) on Days 14, 21 and 28. Group three: intravenous injection of CCP/scrambled siRNA complexes (siRNA concentration of 2 mg/kg) on Days 10, 12, 17, 19 plus an intravenous injection of gemcitabine (10 mg/kg) on Days 14, 21 and 28. Group four: intravenous injection of CCP/PCBP2 siRNA complexes (siRNA concentration of 2 mg/kg) on Days 10, 12, 17, 19 plus an intravenous injection of gemcitabine (10 mg/kg) on Days 14, 21 and 28. Tumor growth in each group was recorded by monitoring the tumor bioluminescence with a Bruker MS FX PRO imaging system (Billerica, CA) on Days 19, 26 and 33. Thirty-four days after inoculation, the mice were euthanized to collect the tumors for subsequent experiments.

Cell apoptosis in tumor tissue was evaluated with a TUNEL assay kit (Invitrogen, Carlsbad, CA) and representative images were captured using a fluorescence microscope (Leica DMI3000B, Germany) and quantified using ImageJ. Tumor sections were subjected to immunohistochemical staining for Ki67 and PCNA to analyze the proliferation of tumor cells. Picrosirius red staining was performed to evaluate the level of collagen in tumor tissues. The tumor sections with immunohistochemical and picrosirius red staining were visualized using an optical microscope and quantified using ImageJ. Hepatic histological study was conducted to evaluate the *in vivo* hepatotoxicity of the treatments as previously reported 30.

## Results

### PCBP2 siRNA reverses the expression of type I collagen in human PSCs and NIH 3T3 fibroblasts

**Figure 1:**
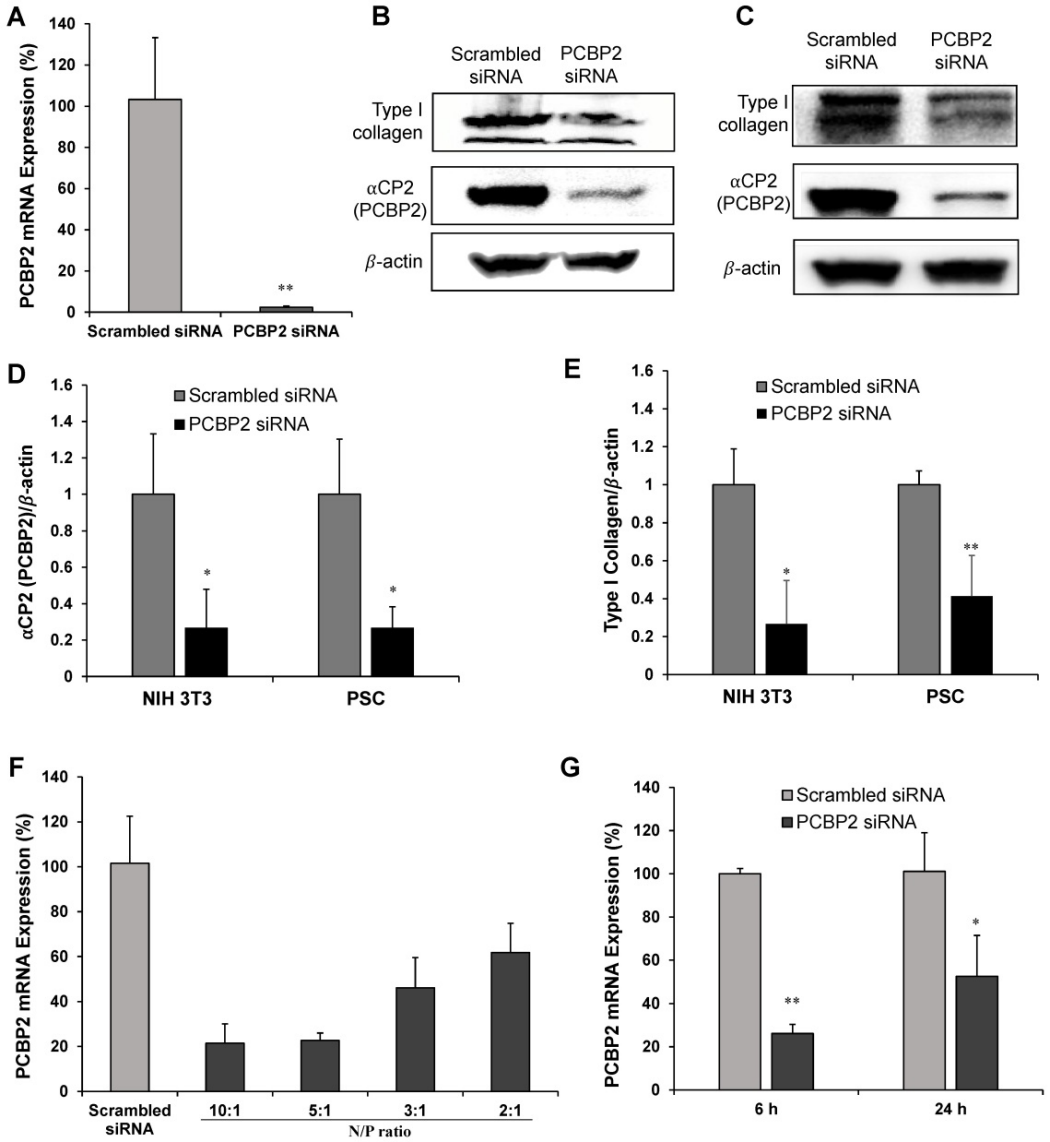
** Silencing activity of the PCBP2 siRNA in human PSCs and NIH 3T3 mouse fibroblasts.** (A) mRNA expression of the PCBP2 gene in human PSCs after incubation with the PCBP2 siRNA condensed with Lipofectamine RNAiMAX for 24 h. Representative western blot images of type I collagen and ⍺CP2 in human PSCs (B) and NIH 3T3 mouse fibroblasts (C) after incubation with the PCBP2 siRNA condensed with Lipofectamine RNAiMAX. Quantitative analysis of ⍺CP2 (D) and type I collagen (E) protein expression using ImageJ. (F) mRNA expression of the PCBP2 gene in human PSCs after incubation with the CP/PCBP2 siRNA nanocomplex at different N/P ratios for 24 h. (G) mRNA expression of the PCBP2 gene in NIH 3T3 cells after incubation with the CP/PCBP2 siRNA nanocomplexes at the N/P ratio 5:1 for 6 and 24 h. All results are presented as the mean ± SD (n = 3). For western blot, three independent experiments were conducted for quantitative analysis. For RT-PCR, total RNA from three independent samples was isolated to measure the silencing effect. (*P < 0.05, **P < 0.01)

Silencing αCP2 with the PCBP2 siRNA was shown to reverse the accumulation of type I collagen in activated HSCs 20, 39, 40. HSCs and PSCs exhibit numerous similarities in their transcriptional phenotypes and share many morphological and functional features. Moreover, both PSCs and HSCs are derived from a common fibroblast lineage and show a distinct susceptibility to the activation by the growth factors TGFβ and PDGF, and cytokines such as TNFα, IL6, IL1, and IL10 23. We, therefore, postulated that silencing PCBP2 would also reverse the pro-fibrogenic effect of PSCs. In the present study, we evaluated the silencing effect of the PCBP2 siRNA on reducing the expression of type I collagen in human PSCs and NIH 3T3 mouse fibroblasts. As shown in **Figure 1A**, the comparison of the levels of the PCBP2 mRNA in human PSCs after transfection with the scrambled siRNA or human PCBP2 siRNA complexed with Lipofectamine RNAiMAX revealed that treatment with the human PCBP2 siRNA resulted in a significant gene silencing effect, with a 97% knockdown of the expression of the PCBP2 mRNA compared to the scrambled siRNA control. Moreover, western blots showed a remarkable decrease in the levels of αCP2 and type I collagen in human PSCs after three transfections with the PCBP2 siRNA (**Figure 1B, 1D-E**). A similar silencing effect of the mouse PCBP2 siRNA was also observed in NIH 3T3 mouse fibroblast cells (**Figure 1C-E**).

The transfection efficiency of cholesteryl peptide siRNA delivery systems into the cells was initially evaluated by incubating the cells with CP/PCBP2 siRNA nanocomplexes at different N/P ratios. As illustrated in **Figure 1F**, at 24 h post-transfection, the expression of the PCBP2 mRNA was reduced as the N/P ratio increased, possibly due to its higher amine content. A significant difference was not observed between the silencing effect at N/P ratios of 10/1 and 5/1, suggesting that a further increase in the N/P ratio did not improve the transfection efficiency. As a result, an N/P ratio of 5/1 was selected for the subsequent experiments with a siRNA loading efficiency of 20.4% (w/w). Furthermore, the silencing effect of the CP/siRNA nanocomplex on the PCBP2 gene was also evaluated in NIH 3T3 mouse fibroblast cells at 6 h and 24 h after the siRNA transfection (**Figure 1G**). A higher gene knockdown efficiency (74%) was observed at 6 h post-transfection, while the silencing efficiency was reduced to 50% at 24 h post-transfection.

### Preparation and characterization of the siRNA nanocomplexes

**Figure 2:**
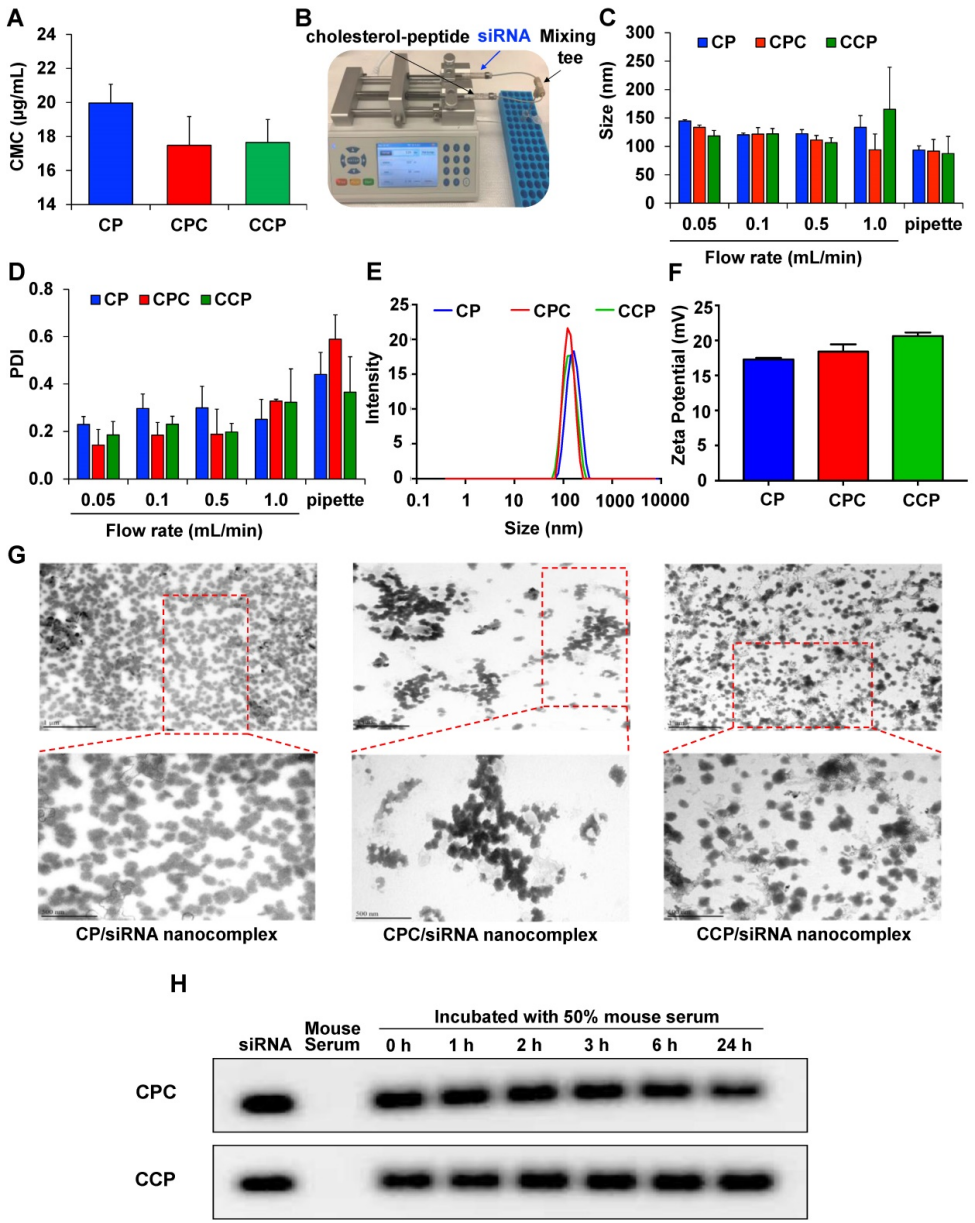
** Characterization of the cholesterol-peptides/PCBP2 siRNA nanocomplex.** (A) CMC values of CP, CPC and CCP micelles. (B) Depiction of the micro-flow mixing device for siRNA nanocomplex preparation. (C) Particle size and (D) PDI of CP, CPC and CCP siRNA nanocomplexes prepared with the micro-flow mixing method at different flow rates. Representative size distribution (E) and zeta potential (F) of CP, CPC and CCP siRNA nanocomplexes prepared with the micro-flow mixing method at a 0.05 mL/min flow rate. (G) Representative TEM images of the siRNA nanocomplexes prepared with the micro-flow mixing method at 0.05 mL/min. The top scale bars represent 1 µm, and the bottom scale bars represent 500 nm. (H) Serum stability of the siRNA nanocomplex in 50% mouse serum. All results are presented as the mean ± SD (n = 3 independent experiments).

All the designed cholesterol-conjugated peptides are amphiphilic molecules. The CMC value of the cholesterol-peptide (CP), cholesterol-peptide-cysteine (CPC), and cholesterol-cysteine-peptide (CCP) conjugates were determined as described before 38. As illustrated in **Figure 2A**, the CMC of CP was 20 μg/mL, while a slightly lower CMC was observed for cysteine modified cholesterol-peptides, with a value of approximately 17.5 μg/mL.

The poor reproducibility and high batch variability of siRNA nanoparticle preparations have largely limited their clinical applications. Currently, “bulk mixing” is commonly used to prepare siRNA nanoparticles through pipetting or vortexing, which lacks the micromixing efficiency and leads to a larger and wider distribution of particle sizes 41, 42. Therefore, the development of effective methods to produce more homogeneous nanoparticles is of paramount importance to ensure the biological performance of siRNAs in the clinic. In the present study, we designed a micro-flow mixing device with two syringes mounted on the same syringe pump to ensure the same flow rate of liquid from the two syringes. As illustrated in **Figure 2B**, two streams are independently loaded with the siRNA and cholesteryl-peptides and mixed thoroughly in a Micro Static Mixing Tee. The flow rate is an important variable affecting the properties of the prepared siRNA nanocomplexes. As shown in **Figure 2C**, slightly larger and more heterogeneous siRNA nanocomplexes were generated when the flow rate was as high as 1 mL/min. Notably, compared with pipetting, PDI values were much lower when the nanocomplexes were prepared with the micro-flow mixing method, indicating that more uniformly sized particles were generated (**Figure 2D**). Furthermore, micro-flow mixing at a rate of 0.05 mL/min produced siRNA nanocomplexes with a narrower size distribution than nanocomplexes produced at a higher flow rate. Thus, an increase in the mixing time (low flow rate) might generate smaller and more homogeneous nanocomplexes, possibly due to the increased ability of cholesteryl-peptides to condense the siRNA by facilitating electrostatic interactions and particle compaction. Therefore, a flow rate of 0.05 mL/min was selected to prepare the cholesteryl-peptide/siRNA nanocomplexes. The z-average particle size of CP, CPC and CCP nanocomplexes prepared by the micro-flow mixing method at a flow rate of 0.05 mL/min were 144.6 nm, 133.4 nm, and 118.3 nm, respectively, and the representative size distribution of each nanopolyplex was shown in **Figure 2E**. The zeta potentials of CP, CPC and CCP/siRNA nanocomplexes prepared at 0.05 mL/min were 17.3 mV, 18.4 mV, and 20.6 mV, respectively (**Figure 2F**).

TEM images of cholesteryl-peptides/siRNA nanocomplexes confirmed the presence of spherical particles and a uniform size distribution (**Figure 2G**). The particle sizes of CP, CPC and CCP siRNA nanocomplexes displayed in the TEM images were slightly smaller than the hydrodynamic diameters detected with DLS because the TEM images were obtained after dehydration.

Naked siRNAs have a very short half-life due to the fast degradation and clearance in the body. Therefore, the ability to protect siRNAs from nuclease degradation in the circulation is the first requirement for an effective systemic siRNA delivery system. Cholesteryl-peptide/siRNA nanocomplexes were incubated with 50% mouse serum for various times (0, 1, 2, 3, 6, or 24 h) to evaluate the ability of the cholesteryl-peptides to increase the serum stability of the siRNA. As illustrated in **Figure 2H**, after a heparin treatment, the gel retardation assay revealed the dissociation of the intact siRNA from CPC or CCP nanocomplexes at 24 h after the incubation, and quantitated in Figure S2, demonstrating that the nanocomplex protected the siRNA from nuclease degradation in the mouse serum for up to 24 h.

### Silencing PCBP2 reverses the expression of type I collagen in NIH 3T3 fibroblasts

**Figure 3:**
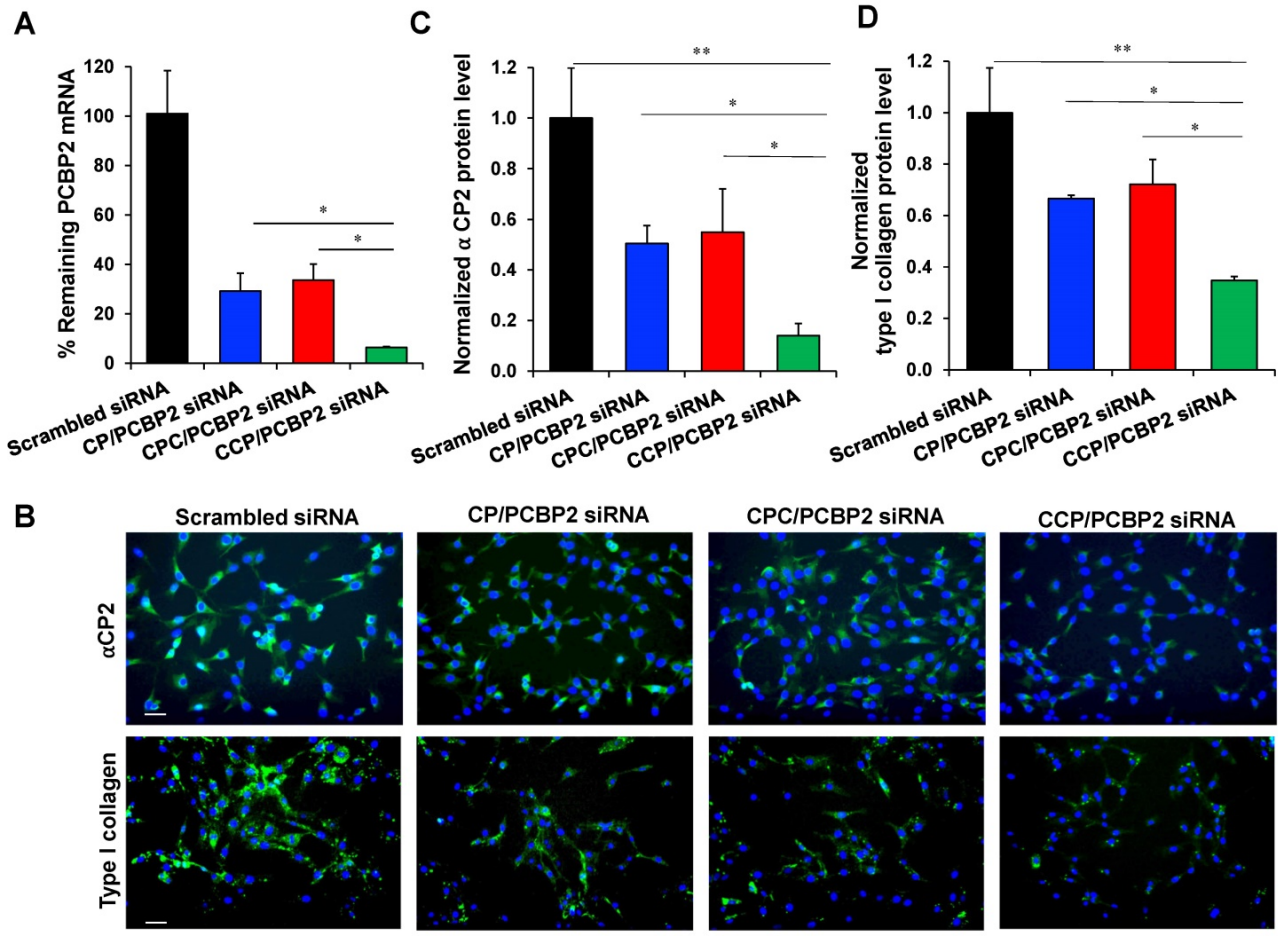
** Silencing activity of the PCBP2 siRNA nanocomplexes in NIH 3T3 mouse fibroblasts.** (A) mRNA expression of the PCBP2 gene in NIH 3T3 mouse fibroblasts after incubation with CP, CPC and CCP/ PCBP2 siRNA nanocomplexes for 6 h. (B) Representative immunostaining images of ⍺CP2 and type I collagen in NIH 3T3 cells after incubation with the CP, CPC and CCP/PCBP2 siRNA nanocomplexes. Scale bars represent 100 μm. Quantitative analysis of ⍺CP2 (C) and type I collagen (D) expressions using image J. The expressions were normalized to scrambled siRNA treated group. All results are presented as the mean ± SD (n = 3 independent experiments). For RT-PCR, total RNA from three independent samples was isolated to measure the silencing effect. For immunostaining, three independent experiments were conducted for each group for quantitative analysis. (*P < 0.05, **P < 0.01)

We next used the optimized micro-flow mixing protocol to prepare PCBP2 siRNA nanocomplexes and evaluated their biological activities. First, NIH 3T3 fibroblasts were incubated with CP, CPC and CCP PCBP2 siRNA nanocomplexes (100 nM siRNA) for 6 h to examine the ability of the nanocomplexes to deliver the siRNA into cells and then trigger gene silencing. As shown in **Figure 3A**, all the PCBP2 siRNA nanocomplexes exerted a significant silencing effect on mRNA expression. Among them, the CCP siRNA nanocomplex exhibited the highest gene silencing activity, while a significant difference in PCBP2 mRNA expression was not observed between the CP siRNA nanocomplex- and CPC siRNA nanocomplex-treated groups. After 6 h of treatment with the CCP siRNA nanocomplex, approximately 94% of gene expression was inhibited, indicating a favorable transfection efficiency. Furthermore, we assayed the ability of the siRNA nanocomplexes to reverse fibrogenesis at the protein level. Images of immunofluorescence staining in NIH 3T3 cells revealed reduced levels of the αCP2 and type I collagen proteins after treatment with the PCBP2 siRNA nanocomplexes (**Figure 3B-D**). Consistent with the real-time PCR results, treatment with the siRNA nanocomplexes decreased the levels of αCP2 and type I collagen, suggesting a reversion of the fibrogenesis. Notably, treatment with the CCP siRNA nanocomplexes produced the most remarkable reduction in αCP2 and type I collagen expression, which were decreased to 14% and 35%, respectively.

### Cellular uptake of siRNA nanocomplexes in NIH 3T3 fibroblasts

**Figure 4:**
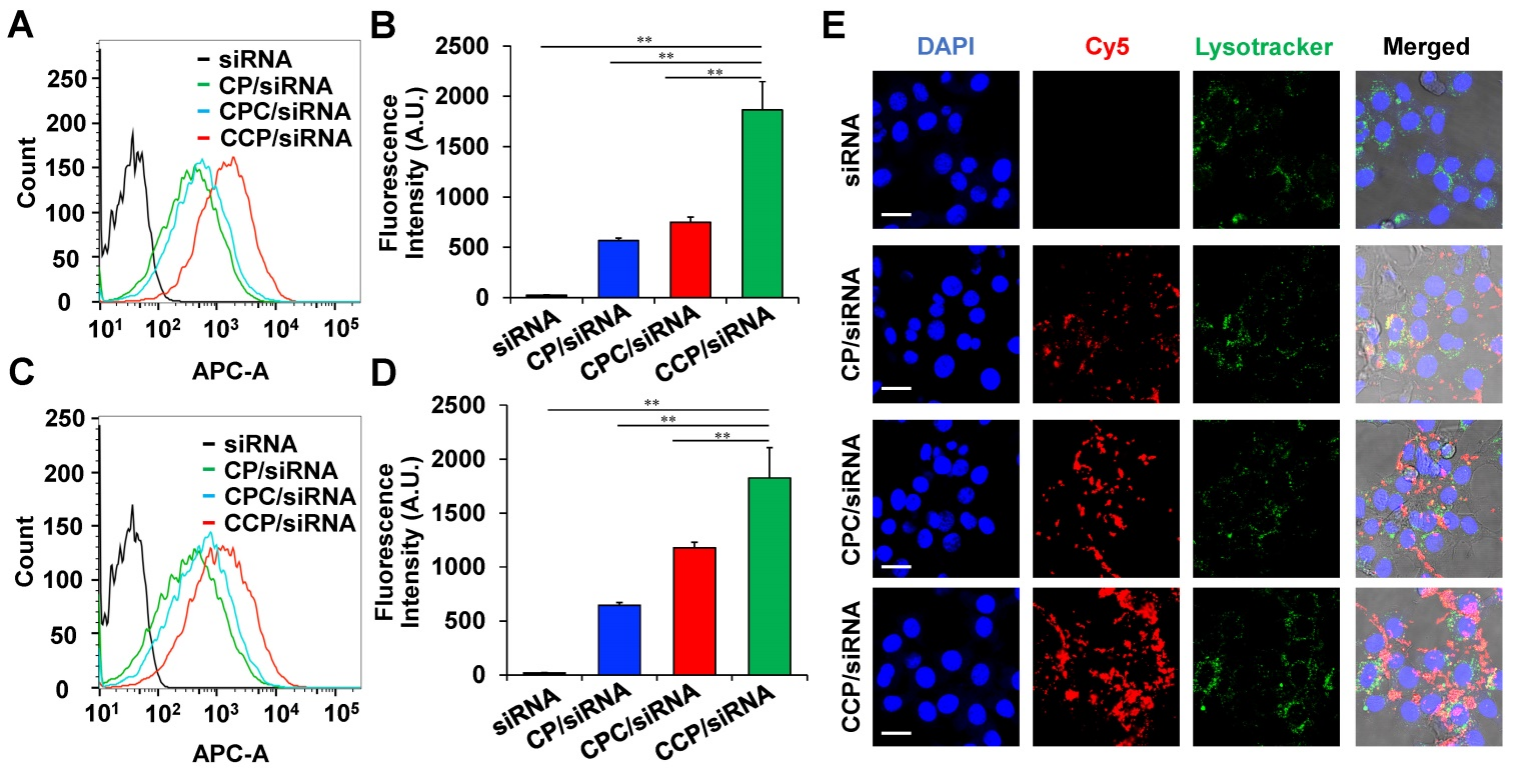
** Cellular uptake of the cholesterol-peptide/siRNA nanocomplexes in NIH3T3 mouse fibroblasts.** Flow cytometry analysis of NIH 3T3 mouse fibroblasts after incubation with free Cy5-siRNA or Cy5-siRNA nanocomplexes for 2 h (A) and 4 h (C). The fluorescence intensity of labeled NIH 3T3 cells at 2 h (B) and 4 h (D). (E) Representative confocal images of NIH 3T3 mouse fibroblasts after incubation with free Cy5-siRNA or Cy5-siRNA nanocomplexes for 4 h. Scale bars represent as 20 μm. All results are presented as the mean ± SD (n = 3 independent experiments). (**P < 0.01)

Cy5-labeled PCBP2 siRNA was used in the study to study the cellular uptake of the CP, CPC, and CCP nanocomplex in NIH 3T3 fibroblasts. Two hours after the transfection, CP, CPC and CCP-based siRNA nanocomplexes exhibited 80.5%, 86.1% and 97.0% cellular uptake in the cells, respectively, while Cy5-siRNA alone only showed negligible uptake (**Figure 4A**). Additionally, the cells treated with CCP siRNA nanocomplex showed a more than 2-fold higher Cy5 fluorescence intensity than cells treated with CP or CPC-based siRNA nanocomplexes (**Figure 4B**). The CCP/Cy5-siRNA nanocomplexes also displayed the highest transfection efficiency and strongest fluorescence signal within the NIH 3T3 cells after a 4 h incubation (**Figure 4C-D**). A confocal microscopy study further supported the flow cytometry data. After a 4 h incubation, the cells were fixed and visualized under the confocal microscope, and the red, green and blue channels indicated Cy5, LysoTracker and DAPI, respectively. The confocal images of NIH 3T3 cells revealed that the cellular uptake of CCP siRNA nanocomplexes was distinguishably higher than CP or CPC-based siRNA nanocomplexes (**Figure 4E**). Based on this result, the cellular uptake of siRNA was facilitated by condensation with CCP-based siRNA nanocomplex.

We also conducted an *in vitro* release study of the siRNA from the nanocomplex. As illustrated in**Figure S3**, cathepsin B can release siRNAs from the nanocomplex, suggesting that the CCP nanocomplex is disrupted after the cleavage of the Val-Cit dipeptide. By contrast, glutathione alone did not release siRNAs from the nanocomplex but enhanced siRNA release in the presence of cathepsin B. This is because that breakdown of the disulfide bond only destabilizes the CCP nanocomplex but cannot dissociate the core-shell structure of the nanocomplex. A combination of glutathione with cathepsin B, however, can have a synergistic effect on the release of siRNAs from the nanocomplex.

**Figure 5:**
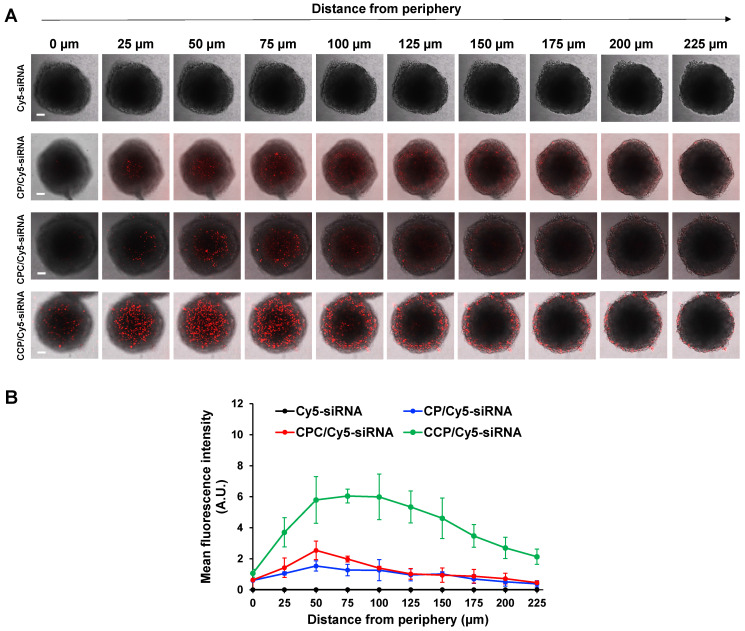
** Penetration in stroma-rich 3D pancreatic cancer spheroids.** The stroma-rich 3D pancreatic cancer spheroids were composed of PANC-1 tumor cells and NIH 3T3 fibroblasts. (A) Representative scanning images of the tumor spheroids with a z-stack of 25 μm after 2 h incubation with free Cy5-siRNA or Cy5-siRNA nanocomplexes. Scale bars represent as 100 μm. (B) Mean fluorescence intensity of free Cy5-siRNA and Cy5-siRNA nanocomplexes in the scanning images vs. the distance from the spheroid periphery. All results are presented as the mean ± SD (n = 3 independent spheroids).

The desmoplastic ECM functions as the major barrier to impede the diffusion and penetration of anticancer therapeutic agents into the tumor tissue. We, therefore, established a stroma-rich 3D tumor spheroid by mixing PANC-1 tumor cells and NIH 3T3 fibroblasts to mimic the desmoplastic characteristics of pancreatic tumor microenvironment 43, 44 and to examine whether the siRNA nanocomplexes can efficiently deliver the siRNA through the dense ECM. The penetration of the siRNA nanocomplexes through the tumor spheroids was monitored by examining the fluorescence intensity of Cy5 with confocal microscopy. As illustrated in **Figure 5A-B**, at two hours post-incubation, very low fluorescence was observed in tumor spheroids treated with free Cy5-siRNA, but tumor spheroids treated with the Cy5-siRNA nanocomplexes showed an obvious fluorescence signal. Notably, the CCP Cy5-siRNA nanocomplex displayed a much higher fluorescence intensity and deeper penetration of Cy5-siRNA in the stroma-rich tumor spheroids compared to CP or CPC-based Cy5-siRNA nanocomplexes. As a result, the CCP siRNA nanocomplex was used for the following studies.

### The PCBP2 siRNA nanocomplex enhances the penetration of small molecules into stroma-rich 3D tumor spheroids

**Figure 6:**
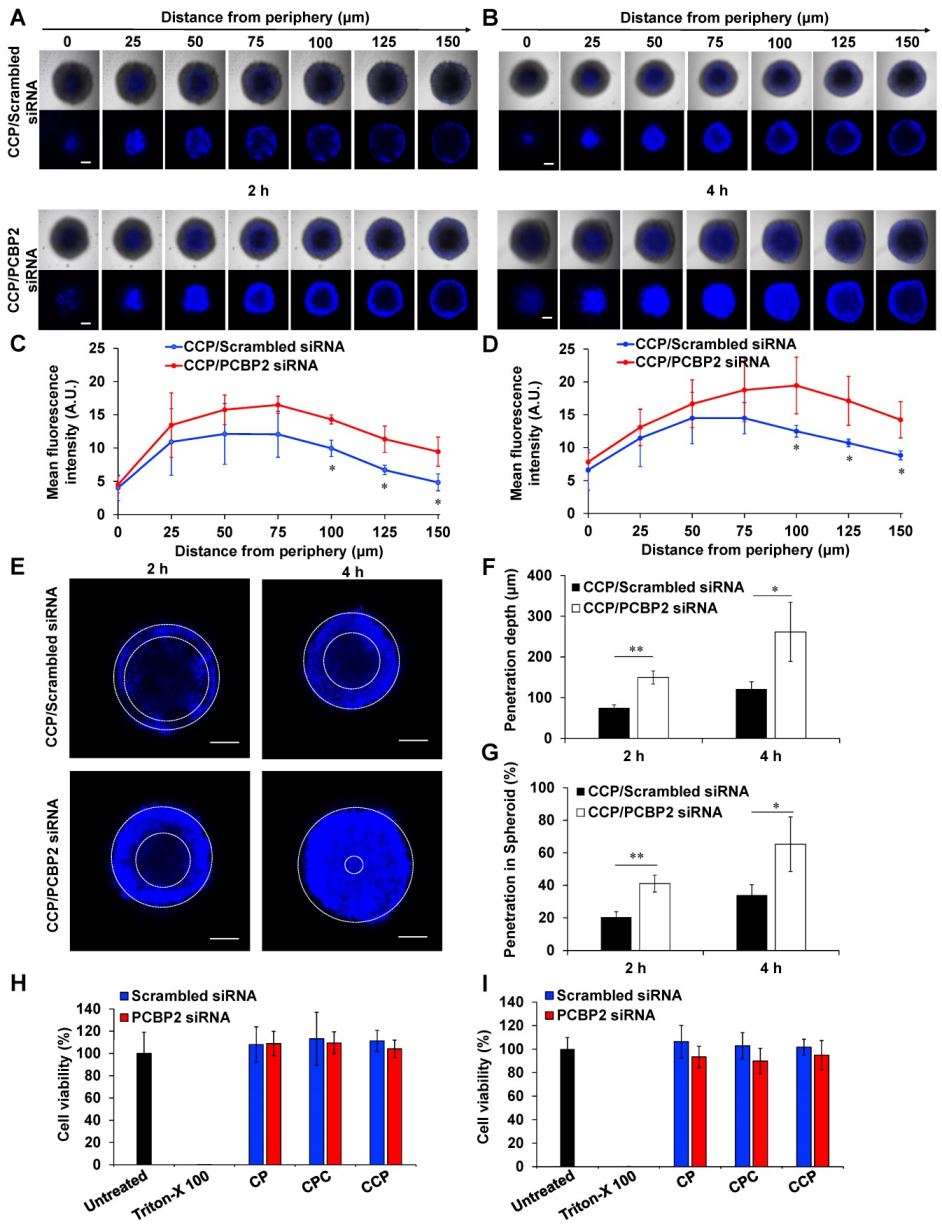
** The CCP/PCBP2 siRNA nanocomplex enhances the penetration of small molecules in stroma-rich pancreatic cancer spheroids and cytotoxicity studies.** The stroma-rich 3D pancreatic tumor spheroids were composed of PANC-1 tumor cells and NIH 3T3 fibroblasts. Representative z-stack confocal images of the stroma-rich tumor spheroids incubated with Hoechst 33258 for 2 h (A) and 4 h (B). Scale bars represent as 200 µm. Mean fluorescence intensity of the z-stack scanning confocal images vs. the distance from the periphery of the spheroids for 2 h (C) and 4 h (D). (E) Representative z-stack images of the stroma rich tumor spheroids at 100 µm from the periphery. Scale bars represent as 200 µm. (F) Quantification of the penetration depth of Hoechst 33258. (G) Percentage of penetration depth of Hoechst 33258 based on the tumor spheroid's size. All results are presented as the mean ± SD (n = 3 independent spheroids). (*P < 0.05, **P < 0.01) Cytotoxicity of NIH 3T3 (H) and PANC-1 (I) cells in 2-D cell culture after incubation with the siRNA nanocomplexes for 24 h. The results are presented as the mean ± SD (n = 5 independent samples).

We have proved that the PCBP2 siRNA can efficiently reverse the accumulation of type I collagen in human PSCs and NIH 3T3 fibroblasts in 2-dimensional cell culture (**Figure 1**). Here, we evaluated whether the CCP/PCBP2 siRNA nanocomplex downregulates stroma and subsequently improves the penetration of small molecule chemotherapy drug in stroma-rich 3D tumor spheroids. We used Hoechst 33258, a widely used fluorescence probe in penetration studies, to mimic small molecule drug in the penetration study. As illustrated in **Figure 6A-B**, the tumor spheroids treated with the CCP/PCBP2 siRNA nanocomplex exhibited a higher fluorescence intensity and deeper penetration of Hoechst 33258 at 2 h and 4 h of incubation than cells treated with the CCP/scrambled siRNA nanocomplex. After a 2 h incubation with Hoechst 33258, a 2-fold higher fluorescence intensity of Hoechst 33258 was observed 150 μm from the periphery in tumor spheroids treated with the CCP/PCBP2 siRNA nanocomplex than in spheroids treated with the CCP/scrambled siRNA nanocomplex, and a 1.6-fold stronger fluorescence signal was observed after a 4 h incubation (**Figure 6C-D**). **Figure 6E** compared the depth of Hoechst 33258 penetration at 100 μm from the periphery in tumor spheroids. Greater than 50% and 90% of the sectional area displayed fluorescence after a 2 h and 4 h incubation with Hoechst 33528, respectively. Treatment with the CCP/PCBP2 siRNA nanocomplex improved the depth of Hoechst 33258 penetration by 1-fold (**Figure 6F-G**). Moreover, the CCP/PCBP2 siRNA nanocomplex does not induce cytotoxicity in PANC-1 tumor cells and NIH 3T3 fibroblasts (**Figure 6H-I**), suggesting that the CCP/PCBP2 siRNA nanocomplex possesses a considerable potential to improve the delivery of small-molecule antitumor drugs in the pancreatic tumor microenvironment by reversing the accumulation of fibrotic stroma. We also compared pharmacokinetics of the CCP/siRNA nanocomplex and free siRNA in mice (**Figure S4**). The free siRNA is degraded and eliminated rapidly from the blood, and only 5% of the dose is left at 15 min post-injection. In contrast, the nanocomplex protected the siRNA from degradation and prolonged its blood circulation up to 24 h. The AUCs of the CCP/siRNA nanocomplex and free siRNA were 4116.3 min•μg/mL and 296.5 min•μg/mL, respectively.

### The PCBP2 siRNA nanocomplex enhances the antitumor activity of gemcitabine in an orthotopic mouse model of desmoplastic pancreatic cancer

**Figure 7:**
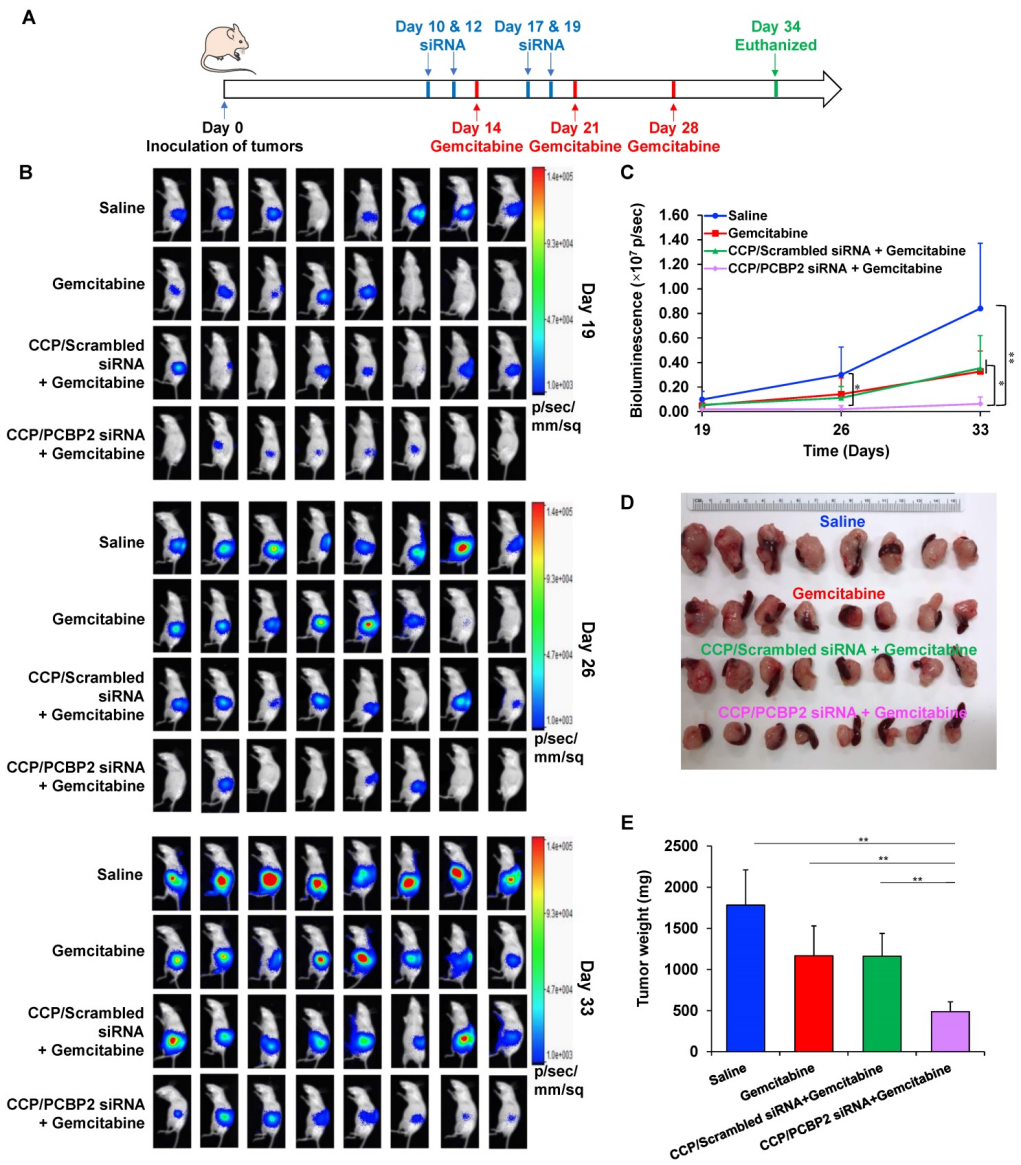
** Antitumor therapeutic efficacy of the CCP/PCBP2 siRNA nanocomplex combined with gemcitabine in an orthotopic mouse model of desmoplastic pancreatic tumor.** (A) Scheme of combination treatment. The desmoplastic orthotopic model was established by co-inoculation of PANC-1/Luc tumor cells with NIH 3T3 fibroblasts into the tail of the pancreas in nude mice on Day 0. The mice received saline, CCP/scrambled siRNA nanocomplex, and CCP/PCBP2 siRNA nanocomplex via tail vein on Days 10, 12, 17, and 19. The mice also received saline or gemcitabine on Days 14, 21, and 28. The mice were euthanized on Day 34 post-implantation. (B) *In vivo* bioluminescence images of mice on Days 19, 26 and 33. (C) Tumor growth curve was determined by the bioluminescence intensity. (D) Images of tumors with spleens. (E) The weight of tumors with spleens. The results are presented as the mean ± SD (n = 8 mice per group). *P < 0.05, **P < 0.01).

**Figure 8:**
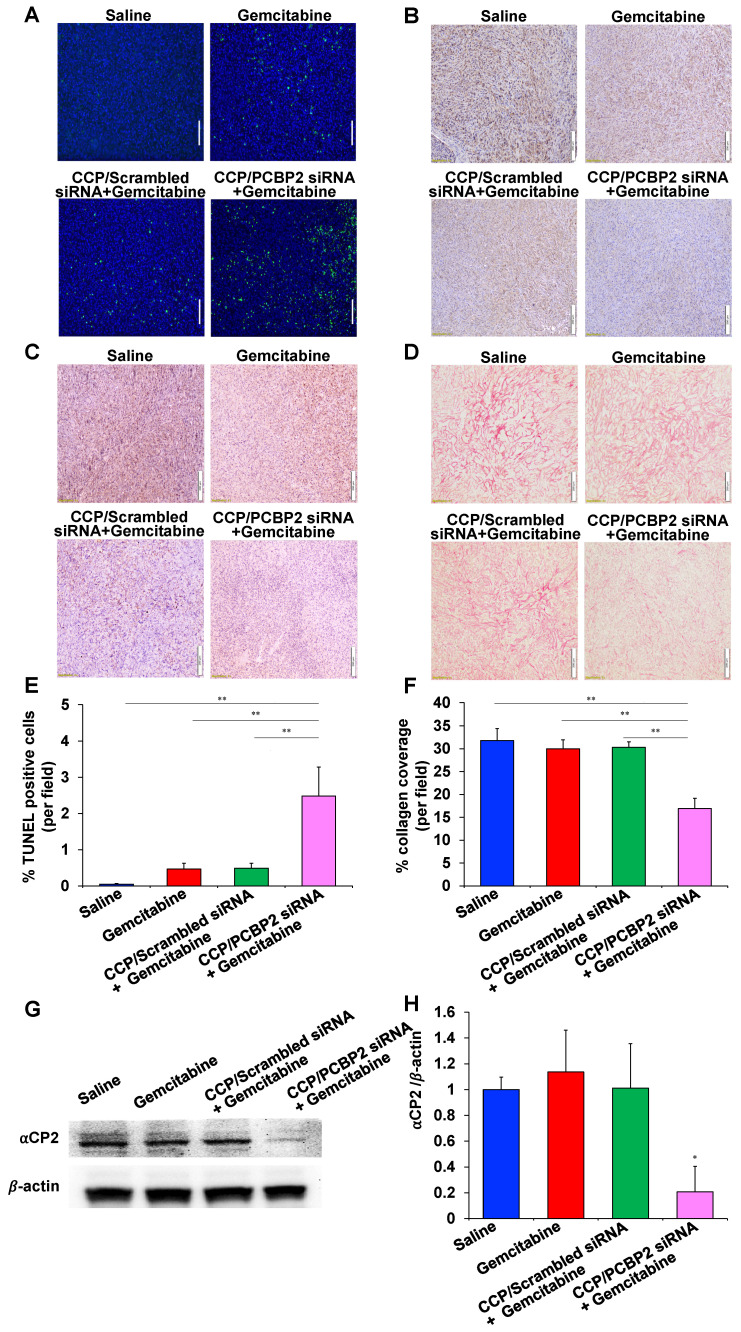
** Histological assessment of tumor tissues.** TUNEL assay (A), Ki67 (B), PCNA (C) immunohistochemistry (IHC) staining and Picrosirius Red (D) staining. The scale bar represents 200 μm. Quantitative analysis of apoptotic cells areas in TUNEL assay (E) and Picrosirius Red stained areas (F) using Image J. The data are presented as the mean ± SD (n = 4 tumors per group, 4 images from each tumor were taken for quantitative analysis). (G) Western blot analysis of ⍺CP2 in tumor tissues. (H) Quantitative analysis of the normalized ⍺CP2 expression. The results are presented as the mean ± SD (n = 3 tumors per group). *P < 0.05; **P < 0.01

The abundant fibrotic stroma is one of the most prominent and unique characteristics of pancreatic cancer, which causes compression and dysfunction of the vasculature, impeding drug penetration and reducing the therapeutic efficacy of antitumor drugs 9. Therefore, beneficial modulation of the fibrotic stroma is a promising strategy to increase the therapeutic efficacy of antitumor drugs 7, 45. Inspired by the potential of the CCP/PCBP2 siRNA nanocomplex to improve the delivery of small-molecule drugs in the desmoplastic tumor microenvironment by reversing the accumulation of fibrotic stroma described above, in this study, we further tested the combinatorial effects of beneficial stroma modulation with chemotherapy in an orthotopic mouse model of stroma-rich pancreatic cancer, which was established by co-inoculating mice with PANC-1/Luc tumor cells mixed with NIH 3T3 fibroblasts (**Figure 7A**). Tumor growth was evaluated by monitoring the bioluminescence intensity of the tumors. Compared with the rapid growth of the tumors treated with saline, gemcitabine alone and the CCP/scrambled siRNA nanocomplex plus gemcitabine treatments resulted in a moderate inhibition of tumor growth. In contrast, treatment with the CCP/PCBP2 siRNA nanocomplex plus gemcitabine significantly inhibited tumor progression compared to the other treatments (**Figure 7B-C**). The weights of harvested tumors were consistent with the bioluminescence results, showing that combination of the CCP/PCBP2 siRNA nanocomplex and gemcitabine chemotherapy resulted in a 72.3% reduction in tumor weights (**Figure 7D-E**). No significant differences in body weight were observed among the four groups, suggesting a good tolerance and low toxicity of the treatments (**Figure S5**). Cell apoptosis in tumor tissues was evaluated using a TUNEL assay. The combination treatment with gemcitabine and the CCP/PCBP2 siRNA nanocomplex showed 5.4-fold higher percentage of apoptotic cells than treatment with gemcitabine alone and the CCP/scrambled siRNA nanocomplex plus gemcitabine (**Figure 8A and 8E**). The IHC staining for Ki67 further confirmed the results of the TUNEL assay (**Figure 8B**). The most significant increase in antitumor efficacy was obtained using the treatment with the CCP/PCBP2 siRNA nanocomplex plus gemcitabine, suggesting the enhanced therapeutic effects of gemcitabine in combination with the CCP/PCBP2 siRNA nanocomplex. To further confirm the antitumor mechanism (blocking DNA synthesis) of gemcitabine when it is used in combination with the CCP/PCBP2 siRNA nanocomplex, we performed IHC staining for PCNA (proliferating cell nuclear antigen), an antigen expressed during DNA synthesis and widely used as a biomarker for proliferating cells. As shown in **Figure 8C**, the lowest PCNA-positive cells were observed after treatment with the CCP/PCBP2 siRNA nanocomplex plus gemcitabine. Meanwhile, no distinct morphology changes were observed in the livers from each group (**Figure S6**), suggesting that there are no significant hepatotoxicity upon the treatment 30.

Picrosirius red staining was performed to study the expression of collagen in the tumor tissue and to explore the mechanism by which the CCP/PCBP2 siRNA nanocomplex improved the antitumor efficacy of gemcitabine. Collagen expression was not significantly reduced in the tumors treated with gemcitabine alone or the CCP/scrambled siRNA nanocomplex plus gemcitabine compared to the saline group. In contrast, the combination of gemcitabine and the CCP/PCBP2 siRNA nanocomplex resulted in the greatest inhibition of collagen expression in the tumor, as it reduced collagen expression by 46.8% (**Figure 8D, 8F**). The expression of the αCP2 protein in the tumors was evaluated using western blot to further confirm the mechanism underlying the reduction in collagen expression. As illustrated in **Figure 8G-H**, levels of the αCP2 protein were significantly decreased by the CCP/PCBP2 siRNA nanocomplex plus gemcitabine treatment, suggesting that the lower density of collagen was mainly due to a post-transcriptional mechanism mediated by silencing the expression of αCP2 with the PCBP2 siRNA. Overall, our data strongly support the hypothesis that a combination treatment with the CCP/PCBP2 siRNA nanocomplex and chemotherapy using gemcitabine should be considered a promising strategy for pancreatic cancer therapy due to its beneficial modulation of the fibrotic stroma.

## Discussion

PDAC is one of the most lethal carcinomas. The activation of PSCs leads to an intense desmoplastic reaction and is a key characteristic of PDAC. The abnormal accumulation of large amounts ECM components, mostly collagen fibers, comprises the dense desmoplasia. Type I collagen is the main component of the ECM 9, 46. According to numerous studies, a high level of type I collagen is responsible for the reduced survival of patients with PDAC by increasing the proliferation and migration of PDAC cells, as well as inducing chemoresistance 8, 9. A western blot assay showed higher expression of type I collagen in fibroblast cells (NIH 3T3) than in tumor cells PANC-1 (**Figure S7**), suggesting that the contribution of collagens in the orthotopic model is predominantly from the fibroblasts.

In the past few years, several preclinical studies have presented appealing results by targeting the tumor fibrotic stroma, including inhibitors targeting the hedgehog signaling pathway, inhibitors target ECM connective tissue growth factor 13, 47, enzymes targeting hyaluronan (HA) 12, 48, and antibodies targeting the hypovascular tumor microenvironment 49. However, the clinical trials targeting the stroma in PDAC have failed 14. Since then, more studies have been conducted to understand the root cause for this failure. Rhim *et al.* deleted Sonic hedgehog (Shh), which drives the formation of desmoplastic stroma, in a mouse model of PDAC. Although Shh-deficient tumors have less amount of stroma, the tumors are surprisingly more aggressive. Further studies demonstrated that Hedgehog-driven stroma inhibits tumor growth partly by suppressing tumor angiogenesis 50. In another study, myofibroblast-depleted tumors showed increased tumor invasion and decreased survival in a transgenic mouse PDAC model. Similarly, PDAC patients with fewer myofibroblasts in their tumors have reduced survival 51. All these studies highlight the complex tumor-promoting and tumor-suppressive functions of the tumor stroma. Therefore, the dense stroma in the tumor microenvironment is a double-edged sword, which acts as not only a drug delivery barrier, but also a protective defense mechanism to restrict tumor progression. The homeostatic restoration of the desmoplastic pancreatic tumor stroma in combination with chemotherapy, targeted therapy, or immunotherapy would be an appealing option for pancreatic cancer treatment. In our previous study, we designed a polymer-drug conjugate to simultaneously deliver a chemotherapeutic agent as well as a transforming growth factor-β (TGF-β) receptor I/II inhibitor, which aims to block the interaction between tumor cells and PSCs. However, the TGF-β pathway plays complex roles in the proliferation and apoptosis of cancer cells. It is demonstrated that TGF-β not only functions as a tumor promotor but also functions as a tumor suppressor in PDAC 52, 53. Therefore, targeting the TGF-β signaling pathway may lead to transformation of normal cells to tumor cells.

Therapeutic approaches with a beneficial modulation, instead of ablation, designed to “normalize” the fibrotic stroma may represent a more attractive and promising strategy for PDAC therapy 45, 46. Collagens are the most abundant components of the ECM and also associated with the major desmoplastic reaction in PDAC 9. For example, type I collagen contributes to the PDAC progression by promoting the proliferation and migration of PDAC tumor cells as well as preventing apoptosis 9, 54. Besides type I collagen, other interstitial collagens such as type III and type IV collagens also have pro-tumorigenic effects. The invasive interaction of the tumor cells with interstitial collagens was associated with disseminated growth of highly malignant PDAC 55. The accumulation of type I collagen in activated HSCs and fibroblast cells is mainly attributed to the increased half-life of its mRNA. Binding of an RNA-binding protein αCP2 (encoded by the PCBP2 gene) to the C-rich region in 3'-UTR of the collagen α1(I) mRNA stabilizes the mRNA and subsequently increases type I collagen expression. Meanwhile, this type of post-transcriptional mechanism has not been observed in quiescent stellate cells 18, 19. Inspired by the fact that PSCs and HSCs are derived from a common fibroblast lineage 23 and our previous superior results showing the effect of silencing PCBP2 on reversing the fibrogenic effect of activated HSCs 20, 38, 40, we expected to reprogram the ECM in PDAC by normalizing the fibrotic stroma through a similar mechanism. To our knowledge, our group is the first to discover the effect of the PCBP2 siRNA on reducing type I collagen level in human PSCs and NIH 3T3 fibroblasts (**Figure 1**). We describe a nano-engineering approach to deliver the PCBP2 siRNA for the homoeostatic restoration of the fibrotic tumor stroma in combination with chemotherapy, leading to increased drug delivery into the tumor microenvironment and killing tumor cells at “home”. In the present study, treatment with the CCP/PCBP2 siRNA nanocomplex improved the penetration of small molecules into the stroma-rich tumor spheroids (**Figure 6**). The stability of nanoparticles is an important factor that influences their penetration in the multicellular tumor spheroids. Micelle stabilized by crosslinking showed better capability to transport their cargos into spheroids. The stabilized micelles exhibit faster and deeper penetration because of their fast exocytosis. By contrast, uncrosslinked micelles have slower exocytosis, which limits their penetration efficiency 56-58. In an orthotopic mouse model of desmoplastic PDAC, the CCP/PCBP2 siRNA nanocomplex dramatically decreased the expressions of αCP2 and type I collagen. Moreover, no significant cytotoxicity was observed in the cells treated with the CCP/PCBP2 siRNA nanocomplex. Therefore, the beneficial modulation induced by the PCBP2 siRNA nanocomplex improved drug penetration into the tumor microenvironment, thus increasing the chemotherapeutic efficacy of gemcitabine *in vivo*.

Since the last century, RNAi has been applied as a powerful research tool, due to its specific knockdown of target gene expression. Although the FDA has approved two siRNA drugs, both of them deliver the siRNA to the liver. Delivery of siRNAs to non-hepatic tissues still remains a challenge. The major obstacle to systemic siRNA application is the lack of an efficient and safe delivery systems. The most desirable siRNA delivery system for enhanced therapeutic efficacy must maintain the structural stability of the siRNA in the circulation and facilitate specific drug release in the target cells 35. Here, the cholesteryl-peptides consisting of lysine and histidine residues were developed as an efficient peptide-based siRNA delivery system. At a neutral pH, the amino group of lysine is positively charged and interacts with the negatively charged siRNA through electrostatic interactions. Due to the protonation of the imidazole group in histidine, the peptides possess a proton sponge activity for endosomal escape. The incorporation of cholesterol into the N-terminus of the peptides enables the formation of a micelle-like structure and effectively increased their charge density to condense siRNAs into nanocomplexes 32. After internalization of the siRNA nanocomplex, we expect the cleavage of cholesterol from peptides to enhance the release of siRNA from the nanocomplex in activated PSCs. Therefore, the Val-Cit dipeptide, a widely used linker in antibody-drug conjugates (ADC), is introduced between cholesterol and the peptide. Our previous study demonstrated the capacity of Val-Cit to improve the release and silencing activity of siRNAs 59. Moreover, Mahajan *et al.* found that the expression of cathepsin B is increased during the activation of PSCs and plays a modulatory role in regulating fibrogenesis and stromal development in PDAC 60. However, the micelle-like nanocarriers always have low structural stability, particularly when injected into the blood, because their nanostructures are difficult to maintain through self-assembly alone 35, 61. Cross-linking of micelles has been recognized as an effective approach to stabilize the self-assembled nanostructure 34. In the present study, we further modified the micellar structure by adding a cysteine into the peptides to stabilize the micellar structure through disulfide cross-linking. Among three cholesteryl-peptide/siRNA delivery systems, the CCP/siRNA nanocomplex exhibited the highest transfection efficiency and strongest silencing activity. This significant improvement might be attributed to the formation of disulfide cross-linked bridges in the micellar structure (**Figure 3-4**). To prove this hypothesis, we performed an Ellman's study using 5,5'-dithio-bis-(2-nitrobenzoic acid) (DNTB) to quantify free thiol groups in CCP and CPC micelles. As shown in **Figure S8**, approximate 20% of free thiol groups in the CCP micelle could be detected, suggesting that 80% of thiol groups formed disulfide bonds. By contrast, only negligible disulfide bonds were formed in the CPC micelle. As illustrated in **Scheme I**, the cysteines in the CCP micelle are closer to the hydrophobic core, leading to a higher chance to form disulfide bonds. Furthermore, the cysteine modification in the peptides facilitates the hydrophobic interaction between lipid moieties, resulting in a more compact and stable complex. Moreover, the more compact nanostructure probably leads to an increased density of positive charges, which will improve the condensation process with nucleic acids 32. The interfacial disulfide cross-linking in the cell-penetrating peptide or polypeptide micelles has been shown to promote stabilization under complex physiological conditions and improve the internalization of peptides into cells through endocytic pathways 34, 62.

Micro-flow preparation decreased the size distribution, produced highly condensed stable nanostructures, reduced the cytotoxicity, and increased the transfection efficiency 41. In an effort to improve the physicochemical properties and biological performance of the cholesterol-peptide/siRNA nanocomplexes, we optimized the preparation process of the nanocomplexes using a micro-flow mixing device. Compared to bulk mixing using a pipette, the micro-flow mixing method resulted in a decrease in the particle size distribution, suggesting that more uniformly sized particles were generated (**Figure 2**). TEM images revealed the nanoscale size and uniform spherical morphology of the siRNA nanocomplexes. Here, the flow rate is an important factor affecting the physicochemical properties of the siRNA nanocomplexes. More homogenous and smaller siRNA nanocomplexes were produced using the micro-flow mixing method when flow rate was as low as 0.05 mL/min, possibly due to the increase in electrostatic interactions between the positively charged peptides and nucleic acid, leading to the production of more compact particles as the mixing time increased.

In conclusion, our results demonstrate the effect of the PCBP2 siRNA on reversing the accumulation of type I collagen in human PSCs and NIH 3T3 fibroblasts. We describe a novel method to condense siRNAs with cholesteryl-peptides, and disulfide cross-linking increases the siRNA transfection efficiency and silencing activity. More homogenous cholesteryl-peptide/siRNA nanocomplexes are produced using the micro-flow mixing method. Treatment with the CCP/PCBP2 siRNA nanocomplex reduces ECM production *in vitro* and* in vivo*, increasing antitumor drug delivery and penetration into the tumor microenvironment. Beneficial modulation of the fibrotic tumor stroma in combination with chemotherapy using gemcitabine significantly inhibits tumor progression in an orthotopic mouse model of desmoplastic PDAC. Overall, our strategy for the homeostatic restoration of the desmoplastic stroma in combination with antitumor therapy represents a novel and promising strategy for PDAC therapy.

### Statistical Analysis

Data were presented as the mean ± standard deviation (SD). The difference between two groups was performed using a two-tailed t-test. Statistical analysis for multiple group comparison was performed using a one-way or two-way analysis of variance (ANOVA) with Tukey's *post hoc* test. A p-value less than 0.05 was considered statically significant.

## Supplementary Material

Supplementary figures.

## Figures and Tables

**Scheme I:**
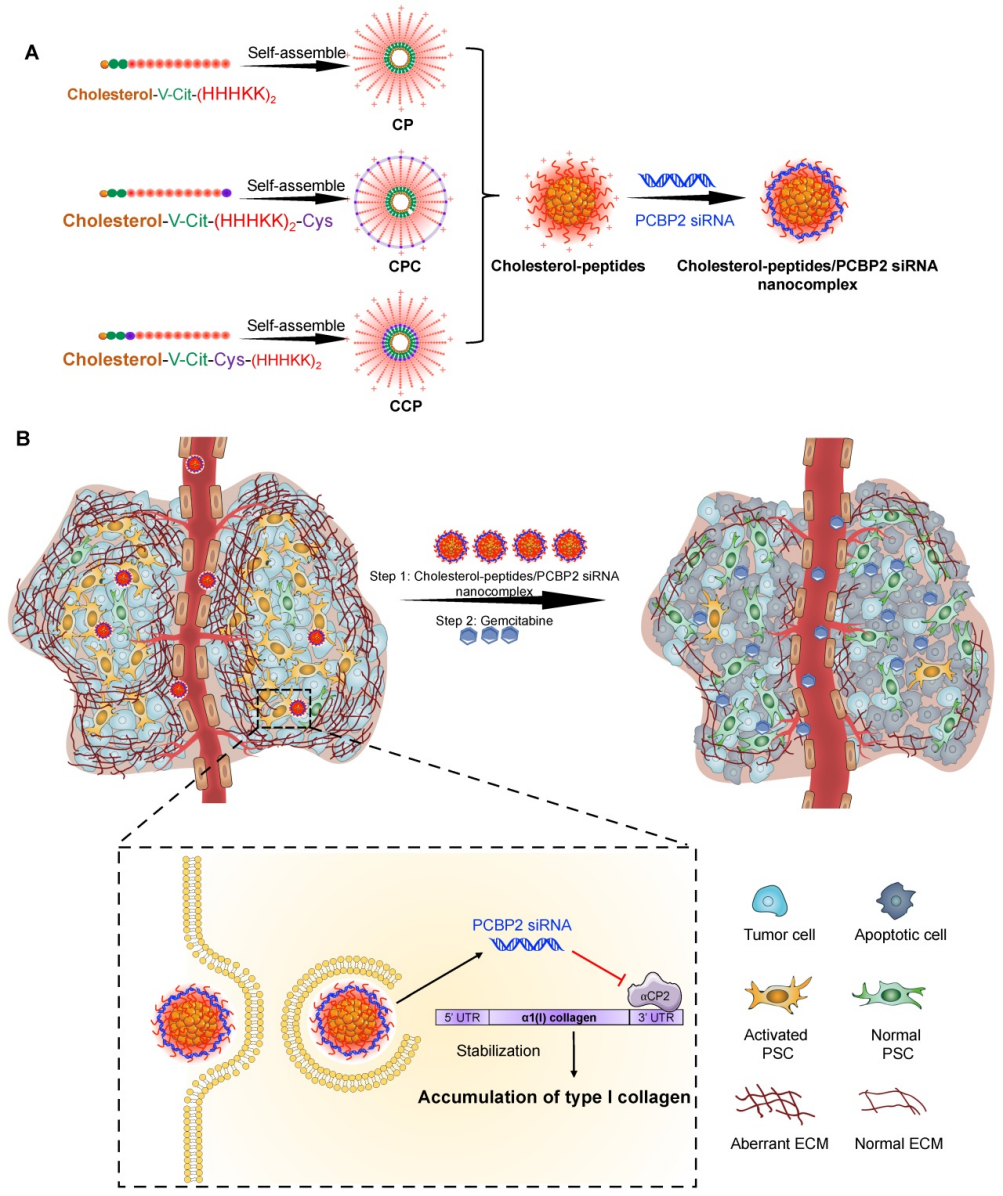
** Schematics of the core-stabilized PCBP2 siRNA nanocomplex and its combination with gemcitabine for PDAC therapy.** (A) Schematics of the cholesterol-peptides/PCBP2 siRNA nanocomplexes. Three types of cholesterol-peptides are used to spontaneously fold into micelle-like particles, followed by condensation with PCBP2 siRNAs using a micro-flow mixing method to form the siRNA nanocomplex. (B) After internalization and release from the endosome in the activated PSCs, the released PCBP2 siRNA blocks the expression of ⍺CP2 and subsequently reverses the accumulation of type I collagen in the PDAC stroma, leading to enhanced antitumor efficiency of gemcitabine.
