# Supplementary material for: Silencing PCBP2 normalizes desmoplastic stroma and improves the antitumor activity of chemotherapy in pancreatic cancer
Source: Theranostics. 2021 Jan 1;11(5):2182–200. doi: 10.7150/thno.53102 (PMC7797682; doi:10.7150/thno.53102)
Supplement: Supplementary file 1 — Supplementary figures. [file thnov11p2182s1.pdf]

Supplementary data

Silencing PCBP2 normalizes desmoplastic stroma and improves the antitumor activity of chemotherapy in pancreatic cancer

Yuanke Li,[1§] Zhen Zhao,[1§] Chien-Yu Lin,[1] Yanli Liu,[1] Kevin F. Staveley-OCarroll,[2] Guangfu Li,[3] Kun Cheng[1,*]

1. Division of Pharmacology and Pharmaceutical Sciences, School of Pharmacy, University of Missouri-Kansas City, 2464 Charlotte Street, Kansas City, MO 64108, USA

2. Department of Surgery, School of Medicine, University of Missouri, One Hospital Drive, Columbia, MO 65212

3. Department of Molecular Microbiology and Immunology, School of Medicine, University of Missouri, One Hospital Drive, Columbia, MO 65212

*Corresponding author:
Kun Cheng, Ph.D.
Curators' Distinguished Professor
Division of Pharmacology and Pharmaceutical Sciences
School of Pharmacy
University of Missouri-Kansas City
2464 Charlotte Street
Kansas City, MO 64108
Phone: (816) 235-2425
Fax: (816) 235-5779
E-mail: chengkun@umkc.edu

[§] Same contribution

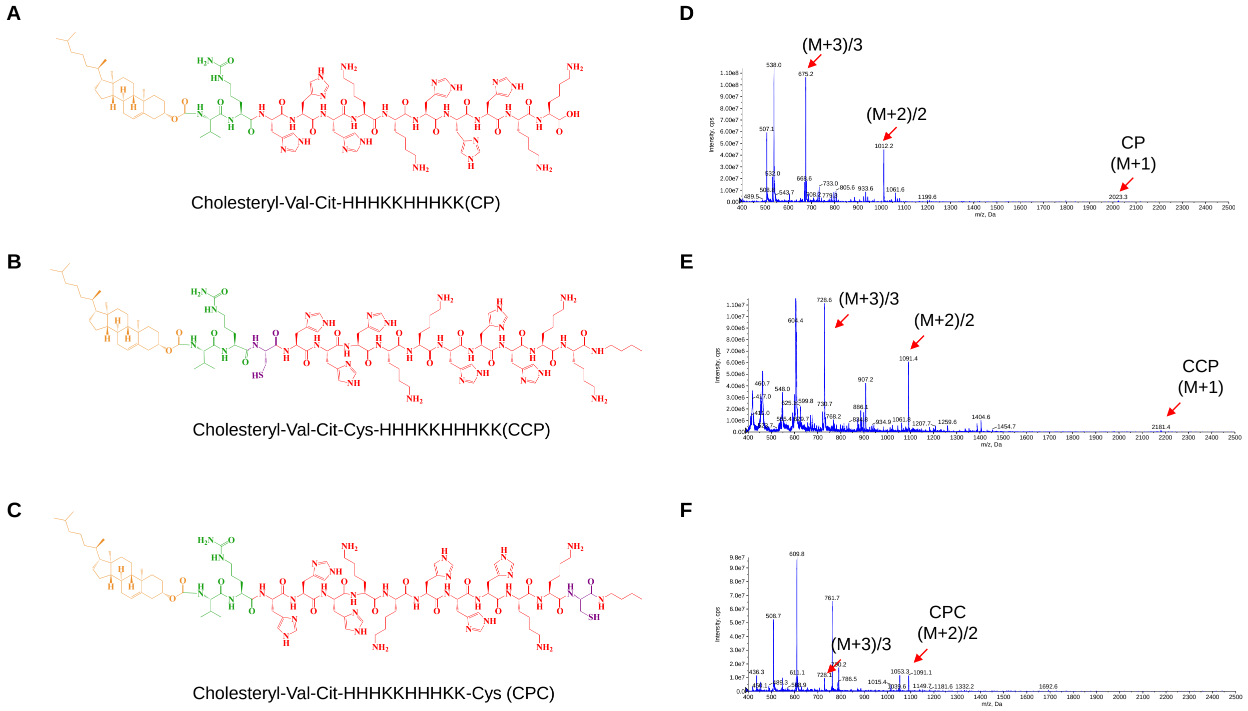

Figure S1. Molecular Structures of CP, CCP & CPC. The structure (A) and mass spectrum (D) of Cholesterol-Val-Cit-HHHKKHHHKK (CP); The structure (B) and mass spectrum (E) of Cholesterol-Val-Cit-Cys-HHHKKHHHKK (CCP); The structure (C) and mass spectrum (F) of Cholesterol-Val-HHHKKHHHKK-Cys (CPC).

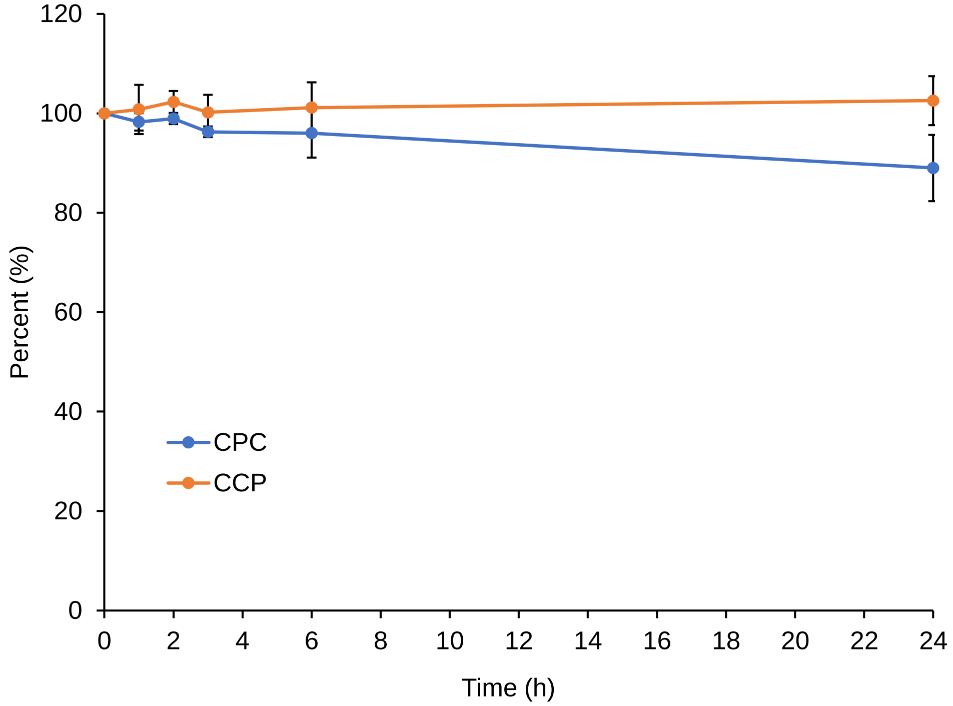

Figure S2. Quantitative measures of serum stability. The serum stability of CCP/siRNA and CPC/siRNA nanocomplexes were performed on 1% agarose gel and quantified using ImageJ. All results are presented as the mean ± SD (n=3 independent experiments).

siRNA	+	−	−	−	−	−	−
CCP/siRNA	−	+	−	+	−	+	+
Glutathione	−	+	+	−	−	−	+
Cathepsin B	−	−	−	+	+	−	+

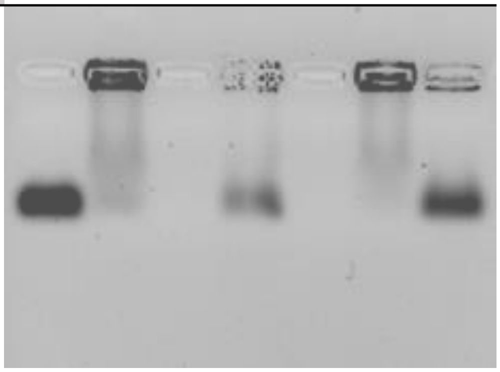

Figure S3. ***In vitro*** **release study of siRNA from the nanocomplex.** The CCP/siRNA nanocomplex was treated with Cathepsin B (12.5 μM) and glutathione (10 mM) at 37 °C for 6 h. Release of the siRNA was determined using a gel retardation assay on a 1% agarose gel. All the experiments were repeated three times.

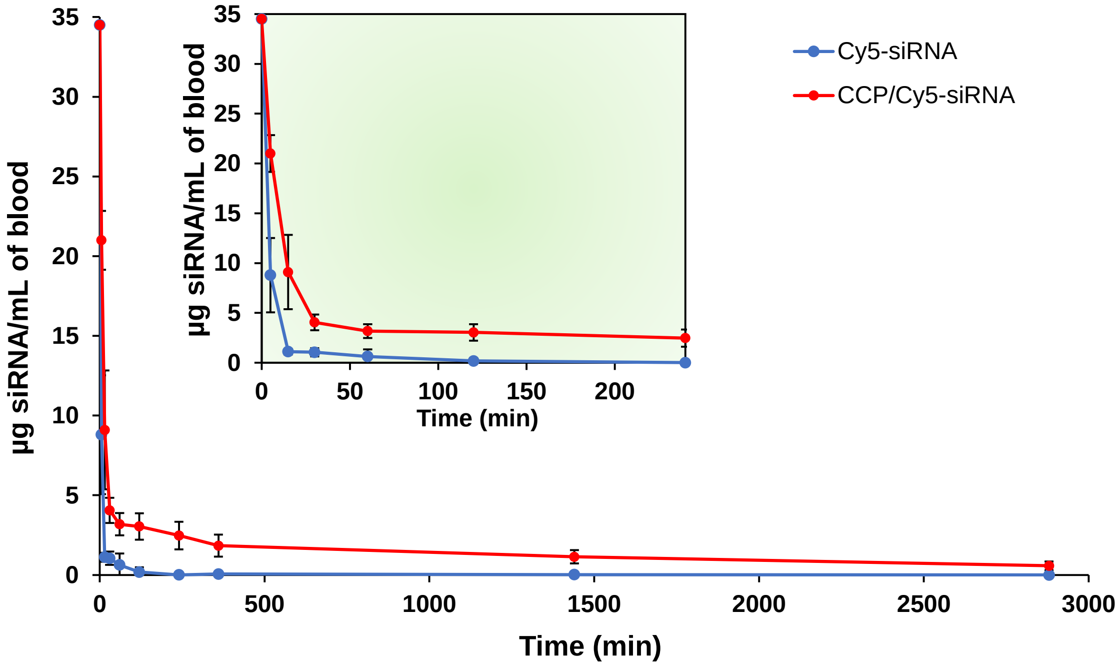

Figure S4. Pharmacokinetic profiles of free siRNA and the CCP/siRNA nanocomplex in mice. The blood (10 μL) was collected from tail vein and mixed with 200 μL lysis buffer at 5 min, 15 min, 30 min, 1 h, 2 h, 4 h, 6 h, 24 h and 48 h post-injection with CCP/Cy5-siRNA or free Cy5-siRNA at a dose of 2 mg siRNA/kg. The fluorescence intensity of Cy5 was measured using a Bruker MS FX PRO imaging system, and the area under the curve (AUC) was calculated. (n=3 mice per group)

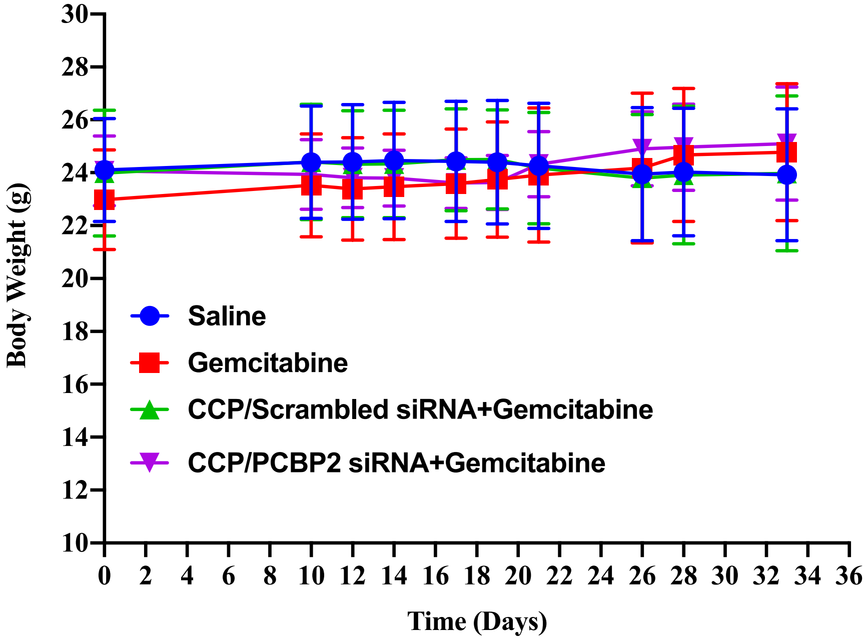

Figure S5. Body weight of the mice treated with different formulations. The body weights of mice were recorded on Day 0, Day 10, Day 12, Day 14, Day 17, Day 19, Day 21, Day 26, Day 28 and Day 33. (n=8 mice per group)

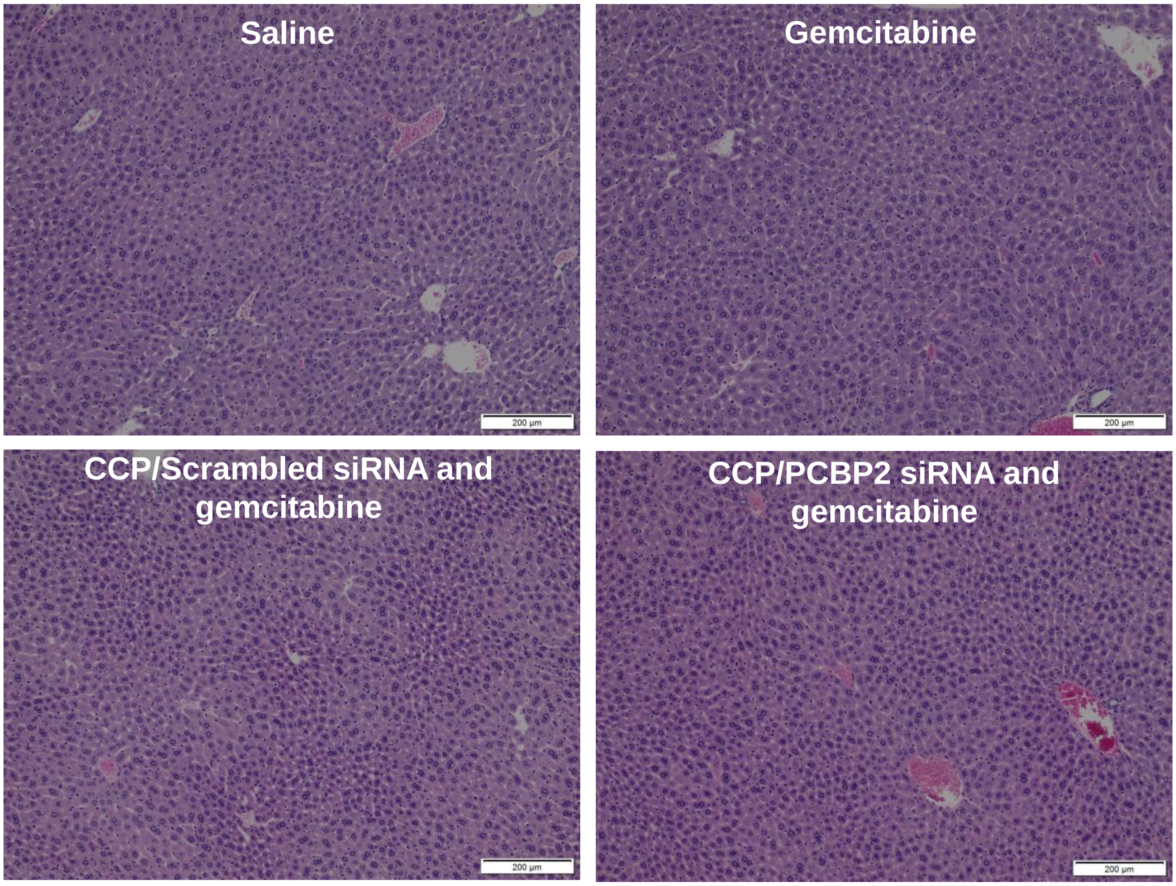

Figure S6. Hepatic histological study. Representative H&E staining images of the livers from the mice (4 mice per group) treated with saline, gemcitabine, CCP/Scrambles siRNA plus gemcitabine, and CCP/PCBP2 siRNA plus gemcitabine. The scale bar represents 200 μm.

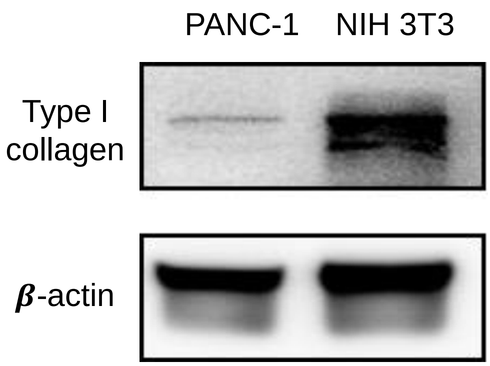

Figure S7. Expression of type I collagen in PANC-1 and NIH 3T3 cells. Expression of type I collagen in the cells was detected using western blot. β-actin was used as an internal control. The experiment was repeated three times.

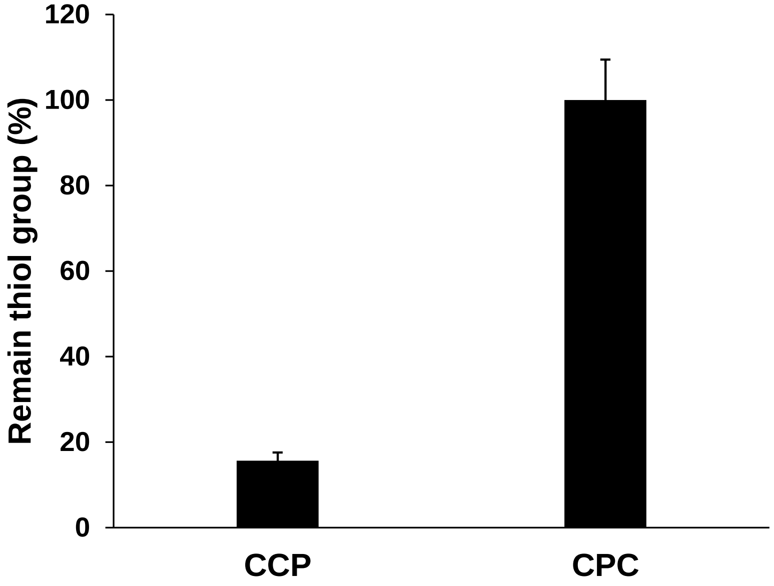

Figure S8. Ellman's study to quantify the free thiol groups in CPC and CCP micelles. Fifty microliter samples were mixed with 950 μL of 0.1 mM DTNB working solution in 0.1 M Tris-HCl (pH7.5). The samples were incubated with DTNB working solution for 2 min, followed by measuring the absorbance at 412 nm. A blank solution was prepared by adding 50 μL of 0.1 M Tris-HCl (pH7.5) to 950 μL of 0.1 mM DTNB working solution. (n=3 independent experiments)
